# OVER SIX TIMES INCREASED RISK OF SUICIDAL IDEATION IN FAMILY CAREGIVERS WITH HISTORY OF CHILDHOOD ABUSE: THE ROLE OF NEUROTICISM

**DOI:** 10.1093/geroni/igae098.2746

**Published:** 2024-12-31

**Authors:** Felipe Jain, Paulina Gutierrez-Ramirez, Miranda Zea, Liliana Ramirez Gomez

**Affiliations:** Depression Clinical and Research Program / Massachusetts General Hospital, Boston, Massachusetts, United States; Massachusetts General Hospital, Boston, Massachusetts, United States; Massachusetts General Hospital, Boston, Massachusetts, United States; Massachusetts General Hospital, Boston, Massachusetts, United States

## Abstract

Despite high reported rates of suicidal ideation (SI) in family caregivers of persons living with dementia, little is known about the relationship between SI and intergenerational trauma. Those with a history of adverse childhood experiences (ACEs) such as neglect and abuse are known to have worse psychological coping skills and higher rates of mental disorders. Personality factors might be mechanistically related to SI in family caregivers, but this has never been tested. We undertook an analysis of the association between intergenerational trauma, measured with the Adverse Childhood Experience (ACE) questionnaire, and a measure of SI from the Quick Inventory of Depressive Symptoms-Self Report, in 81 caregivers of family members living with dementia. We further analyzed whether neuroticism mediated the relationship between ACE and SI. 22% of caregivers exhibited passive SI, and 69% at least 1 ACE. 89% of caregivers with SI reported intergenerational trauma (ACE >= 1), versus 63% of those without SI (p=.04). SI was 4.6x as likely in those with ACE >= 1 than in those without. The odds ratio for SI for those who had experienced childhood neglect was 4.4 (p=.05), and for those reporting childhood abuse was 6.8 (p=.01). Neuroticism significantly mediated the relationship between ACE exposure and SI (p<.05). The risk of SI in family caregivers with history of intergenerational trauma was high in this sample. The relationship between intergenerational trauma, neuroticism, and SI in family caregivers should be further explored and could represent a new treatment target to improve the efficacy of caregiver therapies.

